# Evaluation of Carcinoembryonic Antigen as a Prognostic Marker for Colorectal Cancer Relapse: Insights from Postoperative Surveillance

**DOI:** 10.3390/medsci13040229

**Published:** 2025-10-12

**Authors:** Stefan Titu, Radu Alexandru Ilies, Teodora Mocan, Alexandru Irimie, Vlad Alexandru Gata, Cosmin Ioan Lisencu

**Affiliations:** 1 Faculty of Medicine, “Iuliu Hatieganu” University of Medicine and Pharmacy, 400012 Cluj-Napoca, Romania; stefan.titu@umfcluj.ro; 2 Department of Surgical Oncology, The Oncology Institute “Prof. Dr. Ion Chiricuta”, 400015 Cluj-Napoca, Romania; airimie@umfcluj.ro (A.I.); vlad.gata@umfcluj.ro (V.A.G.); cosminlisencu@yahoo.com (C.I.L.); 3 Department of Physiology, Faculty of Medicine, “Iuliu Hatieganu” University of Medicine and Pharmacy, 400012 Cluj-Napoca, Romania; teodora.mocan@umfcluj.ro; 4 Nanomedicine Department, Regional Institute of Gastroenterology and Hepatology, 400126 Cluj-Napoca, Romania; 5 Department of Oncological Surgery and Gynecological Oncology, “Iuliu Hatieganu” University of Medicine and Pharmacy, 400012 Cluj-Napoca, Romania

**Keywords:** colorectal cancer, carcinoembryonic antigen (CEA), tumor relapse, biomarker, predictive value

## Abstract

**Background/Objectives**: Colorectal cancer (CRC) is a leading cause of cancer-related morbidity and mortality worldwide. This study evaluates the predictive value of Carcinoembryonic Antigen (CEA) in identifying CRC recurrence following surgical resection. **Methods**: This retrospective study was realized in the Oncology Institute in Cluj-Napoca and included 88 patients diagnosed with CRC. Clinical, demographic, and tumor-specific data were collected, including TNM staging, tumor histology. CEA levels were recorded before surgery. Receiver Operating Characteristic (ROC) analysis was performed to determine the diagnostic accuracy of CEA in predicting tumor relapse, and the sensitivity and specificity of various CEA cut-off values were assessed. **Results**: Most patients presented with advanced-stage tumors (T3/T4, 80.6%). CEA levels were significantly higher in patients with lymphatic and perineural invasion and in those with metastases (mean CEA: 45.0 ng/mL for M1 vs. 13.2 ng/mL for M0, *p* = 0.032). ROC analysis revealed an area under the curve (AUC) of 0.877 (95% CI: 0.763–0.949). A CEA cut-off value of 11.73 ng/mL yielded 100% sensitivity and 74.5% specificity for detecting recurrence; **Conclusions**: CEA is a valuable non-invasive biomarker for predicting CRC relapse, with high sensitivity and acceptable specificity. Regular CEA monitoring post-surgery can facilitate early detection of recurrence, improving prognosis.

## 1. Introduction

Colorectal cancer (CRC) is still one of the most common malignancies globally, ranking as the third most frequently diagnosed cancer and the second leading cause of cancer-related deaths worldwide [1]. More than 600,000 deaths are estimated annually due to CRC, underscoring its significant impact on global health. Despite advancements in treatment options, nearly 30% to 50% of patients initially treated for CRC will potentially develop recurrences or metastases following surgery, which not only represents a major challenge for healthcare systems, but also significantly impacts patients’ quality of life and survival outcomes [2].

The prognosis of patients who are diagnosed with CRC is largely influenced by the stage at which the disease is identified. While approximately 75% of cases are diagnosed as localized or regional stages, allowing for potentially curative surgical treatment, the risk of recurrence remains substantial for many patients. This justifies why post-treatment surveillance is necessary, by monitoring any early signs of recurrence or metastasis. Effective postoperative surveillance strategies play a critical role in improving survival rates and treatment outcomes by enabling early detection of relapse. However, the implementation of these strategies presents a few dysfunctionalities, particularly concerning the high costs associated with comprehensive surveillance protocols [3].

Being part of multidisciplinary care, surveillance is the best way to monitor and provide postoperative care to this category of patients with various stages of CRC, being cost-effective [4]. It facilitates early detection of disease recurrence, allowing for earlier initiation of the required treatment and, consequently, improving overall survival rates. Some national and international guidelines, like the American Cancer Society (ACS), National Comprehensive Cancer Network (NCCN), US Multi-Society Task Force (USMSTF), American Society of Clinical Oncology (ASCO), and the European Society of Medical Oncology (ESMO), have suggested multiple strategies regarding postoperative surveillance. Among them, the following are the most relevant: testing of the carcinoembryonic antigen (CEA), colonoscopy, and computed tomography (CT) imaging [2,3,4,5].

In conjunction with imaging studies, serum-based CEA testing is a crucial tool for evaluating the ongoing response to palliative treatments in metastatic cancers. The testing is simple to perform and readily accessible, even in community settings [6].

The Carcinoembryonic Antigen (CEA), discovered in 1965, represents an oncofetal antigen synthesized by epithelial tumor cells and originates from the endoderm [7]. Being a biomarker that is detected through blood sampling, CEA monitoring is both a potentially cheap and safe method, having the advantage of being a non-invasive test used in the follow-up process for colorectal cancer patients [7]. Despite this, results regarding its efficiency in reducing mortality have been controversial. CEA levels can be elevated due to a wide variety of causes, not only malignant but also benign, as well as other habits, such as smoking [8].

According to ESMO guidelines, serum levels of CEA are not sufficient to diagnose colon cancer if a tumor biopsy is absent (because of its low specificity and sensitivity). The marker should be evaluated preoperatively and monitored during the follow-up period to aid in the early detection of metastatic disease. Moreover, the level measurements following colon cancer are of utmost importance because the baseline levels might contribute with information valuable for defining the prognosis of the disease [9]. It is considered that a postoperative serum CEA level exceeding the value of 5 ng/mL (or even >2.35) suggests a worse outcome [10].

Measurement of the blood biomarker levels is recommended as part of the follow-up process to allow for the detection of recurrence of colorectal cancer that might follow primary curative treatment. In colorectal carcinoma, serum carcinoembryonic antigen typically normalizes within 6 weeks post-tumor resection. Persistently elevated CEA levels may reflect the presence of residual neoplastic tissue, either from incomplete resection or recurrence. If CEA remains elevated above baseline, restaging of the malignancy should be considered, as this may indicate disease progression. Levels should not be assessed prematurely, such as 4 to 6 weeks prior to initiating a new therapeutic regimen, as certain chemotherapeutic agents may cause a false elevation [11,12]. Still, there is clinical variation regarding the cut-off level, which serves as a trigger for further investigation [9]. Some authors indicate that CEA monitoring has insufficient sensitivity to be used alone, which is the reason why it should be combined with other diagnostic methods [9]. In addition to this, other authors state that the optimal threshold for serum level investigation decreases over time; the threshold of a single test result should be raised to 10 µg/L. After the second CEA test, the decision of clinicians (for complementary investigations) is recommended to be made based on the progression in time of CEA levels [10].

In the first 3 years after surgery, clinical and CEA assessment is mandatory every 3 to 6 months. Chest and abdominal CT scans are required every 6 to 12 months, while a colonoscopy is recommended every 3 to 5 years, starting 1 year postoperatively. After this period, the follow-up is complete. CEA testing and liver imaging have proven to achieve significant changes in the survival of these patients [3,13].

The adherence to CEA sampling in post-resection colorectal cancer surveillance (having an impact on survival) was defined as having at least 4 test levels measured in a period of 14 months [5]. Overutilization, on the other hand, can lead to serious costs and unnecessary medical visits. The measured adherence to CEA sampling is unacceptably low compared to the guidelines elaborated by NCI-designated Comprehensive Cancer Centers. Therefore, the recommendation is to improve adherence to ensure optimal care for this group of patients [14].

Intensive surveillance may help to detect early recurrence and even to anticipate it, but the survival rate is not as easily influenced by measures like increasing the accuracy and periodicity of screening methods [15,16]. Early recurrence of liver metastases following colorectal cancer is still problematic nowadays, despite the increasing sensitivity of the detection methods, including imaging and laboratory tests. Among the variety of applications that CEA blood sampling has, the detection of liver metastasis recurrence is of high utility. Elevated blood levels of CEA and CA19-9, together with the presence of lymph node metastases and an increased number of liver metastases, have proven to represent risk factors for early recurrence [8,17].

The main objective of this study is to assess the role of CEA as a predictive biomarker for colorectal cancer patients who have presented in our Institute. Moreover, it aims to evaluate the association between biomarker levels and CRC relapse and to identify CEA threshold values that may serve as reliable indicators for early detection of recurrence. An additional objective of the actual study is to investigate the prognostic impact of elevated levels on survival and to explore the contribution of various factors such as age, gender, tumor, stage and histological grade to influence the predictive value of CEA towards CRC relapse.

## 2. Materials and Methods

This study represents a retrospective cohort that included a total of 88 patients diagnosed with colorectal cancer who were treated surgically between 2018 and 2024 at The Oncology Institute “Prof. Dr. Ion Chiricuta”, Cluj-Napoca, Romania. Inclusion criteria consisted of adults (≥18 years) who were diagnosed with histologically confirmed colorectal adenocarcinoma, who underwent surgical resection of the primary tumor with curative intent, and who further enrolled in postoperative surveillance programs (CEA monitoring). Patients with non-colorectal cancers, other malignancies, or incomplete follow-up data, as well as patients who underwent colostomy or did not benefit from curative resection of metastasis were excluded from the study.

The study was approved by the Ethics Committee of “Iuliu Hațieganu” University of Medicine and Pharmacy Cluj-Napoca (No. AVZ247/28 September 2023) and was conducted in accordance with the principles of the Declaration of Helsinki. Written informed consent was obtained from all participants prior to inclusion in the study.

Patient demographic and clinical data were collected from medical records. The following variables were extracted: age, gender, residence (urban or rural), personal and family medical history, symptoms at diagnosis, and details about tumor characteristics, such as TNM staging (T for primary tumor, N for regional lymph nodes, M for distant metastasis), histology, and grading (G1–G3). Pre-treatment clinical data, including imaging results (CT, MRI, ultrasound), and endoscopic findings were also recorded.

All patients were enrolled in a post-operative surveillance program, which included clinical follow-up. The follow-up was performed by a visit to the clinician for clinical examination every 3 to 6 months for the first 3 years, and every 6 months in the 4th and 5th years. Preoperative serum CEA levels were routinely determined within a maximum of 7 days prior to surgical intervention, as part of the standard institutional protocol. Also, yearly, a CT scan of the chest, abdomen, and pelvis was performed (for at least 3 years). Lower digestive endoscopy (LDE) was indicated after a year from the surgical treatment. Part of the patients are still in the follow-up process; however, the first pilot statistical analysis was carried out using records for 1 year postoperative.

As part of routine check-ups, serum CEA levels were measured by using standard enzyme-linked immunosorbent assay (ELISA) techniques. The threshold for an elevated CEA level was considered >5 ng/mL, as it is suggested by the latest clinical guidelines. If any elevated CEA levels were detected, or if clinical signs of recurrence were present, further diagnostic methods such as CT or MRI scans were recommended to assess potential disease recurrence.

Tumor relapse was defined as any recurrence of colorectal cancer, including local recurrence, regional lymph node metastasis, or distant metastasis. Recurrence was confirmed either through histological examination or radiological imaging. Relapse was considered in any existent cases in which the patient exhibited elevated CEA after 6 months from the primary treatment alongside clinical or radiological signs of recurrence.

Descriptive statistics parameters were calculated for all baseline characteristics, including mean, standard deviation, frequency, and percentage. Since testing for normality, performed through Kolmogorov–Smirnov Test resulted in non-normal distribution for CEA parameter, the relation between CEA values and patient demographics, tumor characteristics, and clinical outcomes was assessed using Mann-Witney U test or Kruskall-Wallis Test, where appropriate. The predictive value of CEA for tumor relapse was evaluated using Receiver Operating Characteristic (ROC) curve analysis, using the area under the curve (AUC) to assess its diagnostic performance. Youden index-based calculation of cut-off value for CEA in prediction of CRC local relapse within the first year from intervention was carried out. A *p*-value of <0.05 was considered statistically significant for all tests.

## 3. Results

### 3.1. Analysis of Patient Demographics and Clinical Characteristics

The demographic profile of the study participants highlighted several interesting patterns. A higher proportion of male patients (with the value of 58%) in this cohort was found, with the epidemiological data emphasizing a slight male predilection for colorectal cancer. There was no statistically significant difference between the observed gender distribution, since *p*-value is 0.136. Additionally, the fact that most patients (76.1%) came from rural areas may indicate differences in access to healthcare, lifestyle factors, or environmental exposures. This means the observed residence distribution (23.9% Urban, 76.1% Rural) is significantly different since the *p*-value is lower than 0.05, so the difference was statistically significant. The relatively low frequency of a prior personal cancer history (4.5%) suggests that most patients did not have a genetic predisposition for colorectal cancer.

However, the presence of a prior tumor in another location in 29.5% of patients might suggest shared risk factors, such as age, diet, and smoking history, which could contribute to a higher incidence of secondary malignancies. These demographic characteristics are vital to extensively understanding the context of colorectal cancer incidence and recurrence, which may vary based on geographic, lifestyle, and genetic factors (Table 1).

### 3.2. Clinical Symptoms at Diagnosis and Their Impact

The clinical presentation of colorectal cancer in this cohort shows that the most common symptoms at diagnosis were rectoragy (37.5%) and altered bowel habits (39.8%), which are hallmark signs of this disease. Abdominal pain and anemia were less frequent but present in a significant percentage of patients (15.9% and 6.8%, respectively). This distribution of symptoms emphasizes the importance of early detection, as bleeding and altered bowel transit are often the first signs that require medical consultation. The *p*-value is smaller than 0.05, so the distribution of clinical conditions at presentation is significantly different from an equal distribution.

On the other hand, these symptoms may also be associated with benign conditions, such as hemorrhoids or irritable bowel syndrome, leading to potential delays in diagnosis. In clinical practice, a high index of suspicion is necessary when any of these symptoms is observed, especially in the group of patients with risk factors such as age or a positive family history for colorectal cancer. The need for multi-modal diagnostic approaches, such as CT, MRI, and lower digestive endoscopy (LDE), reinforces the complexity of colorectal cancer diagnosis. A combination of imaging techniques is required to accurately stage the disease (Table 2).

### 3.3. Tumor Staging and Its Clinical Relevance

Tumor localization showed that 23 patients (26.1%) had tumors in the ascending colon, 2 (2.3%) in the transverse colon, 4 (4.5%) in the descending colon, 33 (37.5%) in the sigmoid colon or rectosigmoid junction, and 26 (29.5%) in the rectum.

The TNM staging analysis revealed a significant prevalence of advanced stages at diagnosis, 80.6% of patients presenting with T3 or T4 tumors. Since the *p*-value is far smaller than 0.05, the observed T-stage distribution is significantly different from an equal distribution.

This is consistent with the literature, where a high percentage of colorectal cancers are diagnosed at an advanced stage due to the asymptomatic nature of early disease. The finding that 16% of patients had N2a and N2b nodal involvement further underscores the aggressive behavior of these tumors, so N-stage distribution is significantly different from an equal distribution (*p* < 0.05). Nodal involvement is a critical factor in determining the appropriate strategy of treatment, as the presence of regional lymph node metastasis often indicates the need for adjuvant chemotherapy in addition to the surgical resection. The metastatic rate (5.7%) was relatively low in this cohort, which could be a result of early-stage intervention or the relatively short follow-up period.

Nonetheless, the presence of metastases at diagnosis remains a significant determinant of survival, highlighting the need for more effective strategies regarding early detection and the importance of post-treatment surveillance. CEA marker showed no significant relation with general characteristics, such as gender or residence area. Also, variations across T, N or G categories were not significantly different. However, tumor infiltration as well as the presence of metastases were associated with significantly higher CEA values (Table 2).

### 3.4. Surgical Approaches and Treatment Modalities

Surgical resection was the predominant treatment strategy, as 52.3% of patients underwent this procedure. This is consistent with the current standard of care for localized and resectable colorectal cancer, where surgery remains the cornerstone of treatment. The use of neoadjuvant therapy in 27.3% of patients suggests a significant proportion of cases which were diagnosed at a stage requiring approaches like downstaging before surgery, which is particularly common in rectal cancer. Neoadjuvant therapy, consisting of chemotherapy or radiotherapy, was used to reduce tumor size and improve surgical outcomes, especially in locally advanced cancers.

The variety of surgical techniques employed is justified by the diversity in tumor location and stage. Note that the choice of surgical approach was influenced by factors such as tumor location, size, and the extent of invasion into surrounding tissues, all of which can complicate resection. The widest surgical treatment technique was sigmoid segmental resection or anterior rectal resection (52.27%), followed by right hemicolectomy (20.45%), abdominoperineal resection (12.5%), left hemicolectomy (9.09%), and total colectomy (5.68%). The observed distribution of surgical procedures was significantly different from an equal distribution (*p* < 0.05). Postoperative adjuvant chemotherapy was administered in cases of high-risk tumors, particularly those with lymphatic and perineural involvement, which emphasizes the importance of a multi-disciplinary approach to treatment (Table 3).

### 3.5. Carcinoembryonic Antigen (CEA) as a Prognostic Biomarker

One of the key findings in this study was the role of CEA as a prognostic biomarker for colorectal cancer. While no significant association between CEA levels and basic demographic factors such as gender or residence was found, elevated CEA levels were significantly associated with advanced tumor characteristics, such as metastasis and tumor invasion (either lymphatic or perineural). This supports the growing evidence that CEA can be used as a marker of tumor presence and progression. More specifically, higher CEA values observed in patients with metastatic disease (45.0 ± 76.5) compared to those without metastases (13.2 ± 21.8) confirm its potential as an indicator of disease spread. The association between elevated CEA levels and poor prognosis is also established, demonstrating that CEA testing could offer valuable prognostic information during the follow-up period. CEA marker showed no significant relation with general characteristics, such as gender or residence area. Also, variations across T, N, or G categories were not significantly different. However, tumor infiltration as well as the presence of metastases were associated with significantly higher CEA values (Table 4).

CEA marker showed no significant relation with general characteristics, such as gender or residence area. Also, variations across T, N or G categories were not significantly different. However, tumor infiltration as well as the presence of metastases were associated with significantly higher CEA values (Table 4).

### 3.6. Predictive Value of CEA as a Prognostic Biomarker

**Figure 1:**
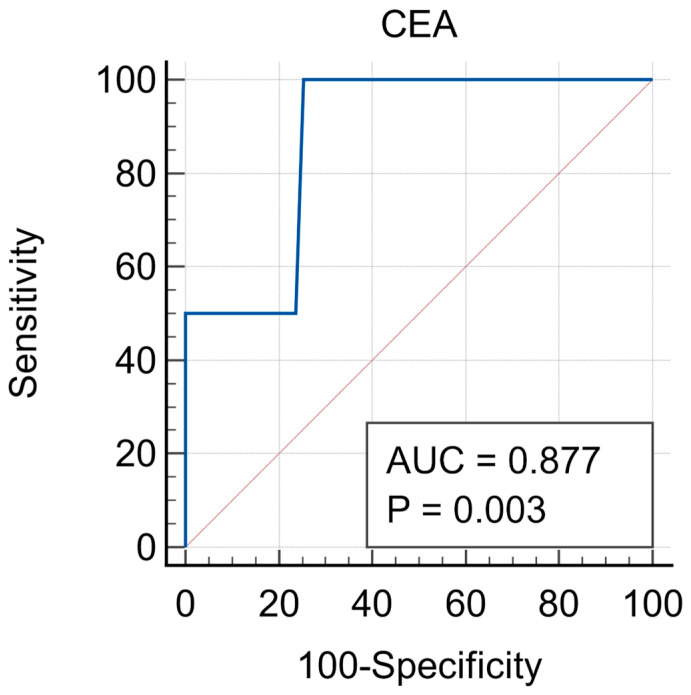
ROC analysis of CEA values in prediction of colorectal tumor relapse within the first year following surgery. The blue line represents the ROC curve for the CEA marker, while the red line indicates the reference line (AUC = 0.5) corresponding to random performance.

The Receiver Operating Characteristic (ROC) analysis provided a detailed evaluation regarding the utility of CEA in predicting tumor relapse. With an AUC of 0.877, CEA demonstrated strong diagnostic accuracy in identifying patients who are at risk of recurrence within the first-year postoperative. This result suggests that CEA can represent an effective non-invasive tool in the surveillance programs, potentially helping clinicians to detect relapse at an early stage, when interventions are more likely to be successful. The cut-off value of 11.73 ng/mL demonstrated 100% sensitivity, meaning that all patients who relapsed had elevated CEA levels. The same value had 74.5% specificity, meaning that most patients without relapse had normal CEA levels. These findings support the use of CEA as a routine marker in postoperative surveillance, particularly in the first few years after surgery, when the risk of recurrence is known to be the highest. (Figure 1).

## 4. Discussion

The predictive capacity of CEA in the evaluation of tumor relapse in colorectal cancer patients is the key finding of this study. The ROC analysis, which is widely used to assess the diagnostic performance of biomarkers, demonstrated highly promising results for CEA to serve as a predictive tool for tumor recurrence, as many other studies have shown. Having an AUC of 0.877 (ranging from 0.763 to 0.949), CEA exhibited a strong diagnostic accuracy, which suggests that it can be a valuable tool for predicting relapse in colorectal cancer patients following primary treatment.

In clinical settings, an early detection of relapse significantly impacts survival rates and the effectiveness of therapeutic interventions. The 100% sensitivity found in this study indicates that CEA levels can reliably identify all the patients who experience tumor recurrence, which is crucial for timely intervention. In other words, none of the patients who relapsed showed a normal CEA level, confirming this biomarker’s ability to detect recurrence when it occurs. This level of sensitivity makes CEA an ideal candidate to be routinely used in post-treatment surveillance, especially due to its non-invasive nature (it can easily be measured by performing blood tests).

However, the specificity of CEA in predicting relapse, which was found to be 74.5%, indicates that while the test is highly sensitive, it also yields false positives in a few cases. This is a common limitation of many biomarkers, especially in the case of a complex disease such as colorectal cancer, where elevated CEA levels can also be associated with other conditions, like benign gastrointestinal diseases, inflammation, or even in the case of smoking [1]. Therefore, while elevated CEA levels are in strong relationship with the presence of recurrence, additional diagnostic methods, such as imaging or histopathological evaluation, should be used to confirm (or infirm) the diagnosis in the cases in which CEA levels are elevated, but the patients do not exhibit clinical symptoms of relapse.

The cut-off value of 11.73 ng/mL for CEA at diagnosis, identified through this study, offers a practical threshold for clinical decision-making. Above this value, the sensitivity and specificity of CEA testing for detecting relapse are both optimized, allowing the healthcare providers to initiate further diagnostic investigations promptly. Establishing a cut-off threshold has a high importance in the current clinical practice, as it helps to differentiate between patients who may require closer follow-up (or additional testing) from those who are less likely to experience relapse in a short term.

Furthermore, the predictive value of CEA for relapse is particularly notable in patients with advanced-stage disease or in those who have already shown the evidence of metastasis. In this cohort, the cases that presented tumor infiltration, particularly lymphatic and perineural involvement, had significantly higher CEA levels, which leads to the fact that more aggressive tumors tend to produce higher concentrations of this biomarker. This relation between CEA levels and tumor characteristics such as invasion and metastasis further support the idea that CEA can be a useful tool in monitoring disease progression and detecting relapse early, before the clinical signs of recurrence become evident.

The study’s findings are limited by the fact that other biomarkers were not investigated. By excluding additional relevant biomarkers, the study may not provide a comprehensive picture of the biological mechanisms underlying the condition being examined. The lack of additional biomarker analysis could affect the study’s generalizability and clinical applicability.

Additionally, it should be noted that several factors, such as smoking, benign gastrointestinal or hepatic conditions, may influence CEA levels and potentially act as confounders. Unfortunately, information regarding these conditions was not consistently available in the medical records of our cohort, which represents a limitation of the current study.

Finally, the relatively small sample size (n = 88) may limit the statistical power and the generalizability of our findings. Also, as all patients were recruited from a single tertiary center, there is a potential for selection bias, which restricts the external validity of the results.

## 5. Conclusions

All in all, this study provides compelling evidence that CEA can play the role of an effective predictive marker for colorectal cancer relapse within the first year after surgery. Its high sensitivity ensures that it can reliably detect recurrences, potentially improving patient outcomes through earlier interventions. The specificity, even though it is not perfect, is still high enough to make CEA a valuable tool in combination with other diagnostic methods. Given its ease of use, non-invasiveness, and strong predictive value, CEA should be considered a key component of surveillance strategies for colorectal cancer patients in the post-operative period. However, further validations in larger cohorts and long-term studies are needed to enhance its applications and to confirm its role in reducing mortality through early detection of relapse.

The use of CEA in routine clinical practice offers several advantages beyond its simplicity and cost-effectiveness. As a blood-based biomarker, it is accessible and can be easily integrated into follow-up protocols, without requiring any advanced technical resources. This makes it especially beneficial for healthcare systems in low- and middle-income regions, where access to more sophisticated imaging technologies might be limited. What is more, incorporating CEA monitoring into regular follow-up visits can enhance the compliance of patients with surveillance programs, as blood tests are generally more acceptable to patients when compared to invasive procedures.

## Figures and Tables

**Table 1:** General characteristics of the patients included in the study.

Parameter	Value (Percent)
Age (mean ± std. Dev)	59.74 ± 11.73
**Gender**	
Male	51 (58.0)
Female	37 (42)
**Residence**	
Urban	21 (23.9)
Rural	67 (76.1)
**Positive personal history**	
Polyposis	2 (2.3)
Diverticulitis	2 (2.3)
Neoplasm with a different localization	26 (29.5)
Other	9 (10.2)
**Positive family history**	
Colon cancer	3 (3.4)
Neoplasm with a different localization	4 (4.5)
Polyposis	2 (2.3)

**Table 2:** Elements of tumor diagnosis.

Parameter	Value (Percent)
**Symptoms**	
Rectoragy	33 (37.5)
Abnormal intestinal transit	35 (39.8)
Abdominal pain	14 (15.9)
Anemia	6 (6.8)
**Pre-therapy paraclinical diagnosis**	
CT	2 (2.3)
CT + LDE	22 (25)
CT/MRI + US + LDE	60 (68.2)
CT/MRI + US + LDE + other	4 (4.5)
**Tumor localization**	
Ascending colon	23(26.13)
Transverse colon	2(2.3)
Descending colon	4(4.5)
Sigmoid colon/rectosigmoid junction	33(37.5)
Rectum	26(29.5)
**TNM staging**	
**T**	
T1	5 (5.7)
T2	12 (13.6)
T3	48 (54.5)
T4	1 (1.1)
T4a	16 (18.2)
T4b	6 (6.8)
**N**	
N1	1 (1.1)
N1a	10 (11.4)
N1b	8 (9.1)
N2a	7 (8)
N2b	7 (8)
Nx	1 (1.1)
**M**	
M0	83 (94.3)
M1	5 (5.7)
**Tumor histology**	
adenocarcinoma	81 (92)
mucinous cancer	6 (6.8)
undifferentiated adenocarcinoma	1 (1.1)
**Grading**	
G1	25 (28.4)
G2	47 (53.4)
G3	16 (18.2)
**Tumor Invasion**	
Lymphatic	17 (19.3)
Lymphatic and perineural	20 (22.7)

**Table 3:** Surgical Treatment and Neoadjuvant Treatment.

Parameter	Value (Percent)
Neo-adjuvant treatment	24 (27.3)
Surgical technique	
Total colectomy	5 (5.68)
Sigmoid segmental resection or anterior rectal resection	46 (52.27)
Abdominoperineal resection	11 (12.5)
Left hemicolectomy	8 (9.09)
Right hemicolectomy	18 (20.45)

**Table 4:** The association between CEA levels and clinical parameters in colorectal cancer.

Parameter	CEA Value (Mean ± std.dev)	*p* Value
**Gender (no, percent)**		0.287
Male	20.5 ± 36.2	
Female	8.7 ± 11.8	
**Residence (no, percent)**		0.583
Urban	23.9 ± 33.7	
Rural	13.1 ± 27.3	
**Symptoms**		0.538
Rectoragy, Abdominal Pain or Abnormal Intestinal transit	16.4 ± 30.0	
Weight loss or Anemia	5.8 ± 4.6	
**T**		0.752
T1	3.7 ± 4.1	
T2	6.4 ± 8.7	
T3	19.3 ± 34.9	
T4a	11.6 ± 11.4	
T4a	6.7 ± 4.6	
**N**		0.229
N1a	7.8 ± 7.8	
N1b	41.4 ± 65.5	
N2a	8.5 ± 5.1	
N2b	14.0 ± 9.3	
x	7.0 ± 7.0	
**M**		0.032
M0	13.2 ± 21.8	
M1	45.0 ± 76.5	
**G**		0.807
G0	11.43 ± 23.6	
G1	17.4 ± 33.3	
G2	15.8 ± 22.9	
**Tumor invasion**		0.024
Absent	8.2 ± 13.9	
Lymphatic	13.3 ± 17.1	
Lymphatic and perineural	33.0 ± 48.7	

## Data Availability

The original contributions presented in this study are included in the article. Further inquiries can be directed to the corresponding author.

## Abbreviations

The following abbreviations are used in this manuscript:

CRC	Colorectal cancer
CEA	Carcinoembryonic Antigen
ROC	Receiver Operating Characteristic
AUC	Area Under the Curve
ACS	American Cancer Society
NCCN	National Comprehensive Cancer Network
USMSTF	US Multi-Society Task Force
ASCO	American Society of Clinical Oncology
ESMO	European Society of Medical Oncology
CT	Computed Tomography
MRI	Magnetic Resonance Imaging
ELISA	Enzyme-linked immunosorbent assay
LDE	Lower Digestive Endoscopy
